# The impact of national comprehensive medical reform on healthcare quality: evidence from China

**DOI:** 10.3389/fpubh.2026.1866596

**Published:** 2026-07-15

**Authors:** Weixiang Pan, Qi Chen, Zulei Qin, Fang Wang

**Affiliations:** 1 Institute of Medical Information and Library, Chinese Academy of Medical Sciences and Peking Union Medical College, Beijing, China; 2 School of Management, Shanghai University, Shanghai, China; 3 School of Health Policy and Management, Chinese Academy of Medical Sciences and Peking Union Medical College, Beijing, China

**Keywords:** China, difference-in-differences, healthcare quality, national comprehensive medical reform, patient safety

## Abstract

**Introduction:**

Healthcare quality is an important dimension of health system performance and a key criterion for evaluating the effectiveness of China’s deepening healthcare system reform. This study treats the National Comprehensive Medical Reform pilot as a quasi-natural experiment to examine its impact on healthcare quality and further analyzes the potential mechanism of pharmaceutical pricing reform.

**Methods:**

Based on panel data from 31 provinces in China from 2012 to 2020, combined with data on medical malpractice liability disputes from China Judgments Online, this study employs a multi-period difference-in-differences model to identify the policy effect of the National Comprehensive Medical Reform pilot. Healthcare quality is measured by the incidence of medical malpractice liability disputes and inpatient mortality, and the mechanism of pharmaceutical pricing reform is analyzed through changes in the structure of medical expenditures.

**Results:**

The study finds that the National Comprehensive Medical Reform pilot significantly reduced the incidence of medical malpractice liability disputes and inpatient mortality, indicating that the reform helped improve healthcare quality. Mechanism analysis shows that the reform significantly reduced drug expenditure per outpatient visit and per inpatient admission, lowered the share of drug expenditure, and increased the share of medical service fees. These findings suggest that the National Comprehensive Medical Reform may have improved healthcare quality by reducing hospitals’ dependence on drug-related revenue, increasing the relative importance of service-based income, and reshaping hospital revenue structures and provider incentive mechanisms. However, the share of examination fees increased significantly, indicating the possible presence of substitute revenue-seeking incentives and associated quality risks during the reform process.

**Discussion:**

The National Comprehensive Medical Reform pilot had a positive effect on improving healthcare quality, and its mechanism may be related to hospital revenue restructuring and the optimization of healthcare service incentives induced by pharmaceutical pricing reform. Future reforms should further strengthen coordination among medical care, medical insurance, and pharmaceuticals, continue to optimize medical service pricing and compensation mechanisms, and curb substitute revenue-seeking behavior, so as to provide a more stable and sustainable institutional foundation for the continuous improvement of healthcare quality.

## 1 Introduction

Healthcare quality is a central dimension of health system performance and a key link connecting resource inputs, care delivery processes, and population health outcomes (1). Health system reform aims not only to expand access to and coverage of medical services, but also to ensure that patients receive care that is safe, effective, timely, and patient-centered (2, 3). Poor-quality care, by contrast, has become a major constraint on further health improvement. Existing evidence suggests that, among deaths amenable to healthcare, the health losses attributable to poor-quality care exceed those caused by insufficient use of health services alone (4). In China, this challenge has become increasingly salient, particularly in the form of patient safety risks and adverse events during care delivery. Between 2012 and 2017, 36,498 patient safety incidents were reported nationwide, with medication-related, nursing-related, and surgery-related events being the most common (5). A medical record review of Chinese general hospitals further showed that more than one-fifth of adult inpatients experienced at least one adverse event resulting in temporary harm, with surgery-related and medication-related problems identified as the leading causes (6).

Since the launch of the new round of healthcare reform in 2009, China has made substantial progress in increasing the supply of medical services, expanding basic medical insurance coverage, and improving access to care, thereby helping to alleviate, at least to some extent, the long-standing problems of “difficulty in seeing a doctor” and “expensive medical care” (7, 8). In October 2016, the State Council issued the Healthy China 2030 Plan Outline, which explicitly called for a shift from a treatment-centered approach to a model centered on people’s health. This policy orientation was further reinforced in September 2020, when improving the quality and service level of healthcare services was identified as a key task. Against this background, the priorities of China’s healthcare reform have gradually shifted from expanding service coverage to the continuous improvement of healthcare quality (9).

To deepen these reforms, the Leading Group for Healthcare Reform of the State Council launched two batches of National Comprehensive Medical Reform (NCMR) pilot provinces in 2015 and 2016. The pilot program encouraged these provinces to explore innovative approaches and develop reform models that could be replicated and promoted elsewhere, with the aim of advancing the new round of healthcare reform. The first batch of NCMR pilot provinces, announced in February 2015, included Jiangsu, Anhui, Fujian, and Qinghai. In May 2016, the second batch expanded the program to seven additional provinces and municipalities, including Shanghai, Zhejiang, Hunan, Chongqing, Sichuan, Shaanxi, and Ningxia.

Although the National Comprehensive Medical Reform (NCMR) pilot has been implemented for several years, empirical evidence on its effects remains limited. Early studies mainly relied on case analyses and local experience summaries, lacking systematic evaluations based on nationwide data (10). Later research, often using difference-in-differences designs, has focused primarily on whether the NCMR alleviated the problems of “difficulty in accessing medical care” and “high medical costs.” Existing evidence suggests that the reform reduced hospital operating costs, strengthened expenditure constraints, standardized clinical processes, and lowered patients’ medical spending, although its effects on service capacity and outpatient expenditures remain mixed (11). Micro-level studies further indicate that the NCMR reduced out-of-pocket spending, improved self-reported health and patient satisfaction, increased healthcare utilization, expanded insurance coverage, and encouraged care-seeking at lower-level hospitals (12). From the perspective of resource allocation, the reform also improved the efficiency of regional medical resource allocation, although regional and group heterogeneity persisted (13).

Overall, existing studies have provided a relatively comprehensive account of the effects and mechanisms of the NCMR in reducing medical burden, improving access to care, and promoting healthcare utilization from both macro- and micro-level perspectives. Their findings are broadly consistent in showing that the reform has helped alleviate the problems of “difficulty in accessing medical care” and “high medical costs.” By contrast, to our knowledge, direct evidence on the impact of the NCMR on healthcare quality remains lacking. Only a small number of studies have examined this issue indirectly through outcomes such as patient satisfaction and institutional service capacity, and their findings remain mixed.

Against this background, we treat the NCMR pilot policy as a representative quasi-natural experiment. Drawing on panel data for 31 provinces in China from 2012 to 2020 and provincial data on medical malpractice dispute cases obtained from China Judgments Online, we apply a multi-period difference-in-differences (DID) model to identify the causal effect of the NCMR pilot policy on healthcare quality.

## 2 Policy background and hypotheses

During the implementation of the NCMR pilot, the pilot provinces introduced a series of reforms under the framework of coordinated action across medical care, medical insurance, and pharmaceuticals. These measures covered multiple areas, including reforms to the pharmaceutical supply system, adjustments to medical service prices, reconstruction of the compensation mechanism for public hospitals, and reform of provider payment methods (14, 15). Although reform pathways and policy details varied across regions, pharmaceutical pricing reform, initiated through drug-related reforms and supported by adjustments to medical service prices, constituted a core component commonly advanced across most pilot provinces (16). Compared with locally specific initiatives that were implemented only in a limited number of regions, these reform measures were more generalizable and were more likely to exert sustained effects on hospitals’ clinical behavior and healthcare quality by reshaping hospital revenue structures and the incentive mechanisms faced by medical staff (17, 18). Accordingly, this study examines pharmaceutical pricing reform as a potential mechanism linking the NCMR pilot to healthcare quality.

More specifically, on the one hand, the NCMR pilot sought to reduce inflated prices of drugs and high-value medical consumables and to weaken hospitals’ dependence on pharmaceutical sales revenue by abolishing drug markups, promoting centralized procurement, compressing unreasonable markups in circulation, and strengthening medical insurance and financial supervision. On the other hand, it gradually established new compensation and incentive mechanisms through fiscal compensation, performance management, and adjustments to the structure of medical service prices. In this process, zero-markup drug policies and volume-based procurement helped weaken the link between medical revenue and drug sales, thereby mitigating the distorted incentives associated with “profit-making through drug sales.” This may reduce inappropriate prescribing and unnecessary treatment and encourage hospitals to place greater emphasis on standardized and patient-centered care (19–21). At the same time, fiscal compensation and related governance measures may help reduce the risk that hospitals, after losing drug-related revenue, shift toward alternative profit-seeking behaviors through excessive testing or greater use of consumables, thereby providing institutional support for maintaining the public welfare orientation and stable operation of hospitals (22).

On this basis, the pilot provinces further promoted structural adjustments in medical service prices. While compressing unreasonable expenditures on drugs, examinations, and consumables, they gradually increased the prices of services that better reflect the technical labor value of medical staff, such as consultation, nursing, and surgery. Such adjustments may reshape hospital revenue structures and provider incentives, encouraging hospital operations and physicians’ behavior to move away from dependence on drug sales and diagnostic testing and toward greater emphasis on technical service value and care quality (23).

Based on the above analysis, this study proposes the following hypothesis:

H1: Pharmaceutical pricing reform may serve as a potential mechanism through which the NCMR pilot improves healthcare quality by optimizing the structure of medical expenditures.

## 3 Materials and methods

### 3.1 Variables and data sources

This study uses panel data for 31 provinces in China from 2012 to 2020 to evaluate the policy effects of the NCMR pilot launched in 2015 and 2016. The data are mainly drawn from *China Judgments Online*, the *China Health and Family Planning Statistical Yearbook*, the *China Health Statistics Yearbook*, and the *Statistical Yearbooks* of individual provinces.

*China Judgments Online* provides one of the key indicators used in this study to measure healthcare quality. On January 1, 2014, the Supreme People’s Court of China officially implemented the Provisions on the Publication of Judgment Documents by People’s Courts on the Internet. According to these provisions, all effective judgment documents issued by courts at different levels and in different regions should be published online, except for five categories of cases, including those involving state secrets, personal privacy, or crimes committed by minors. Given the substantial incompleteness of judicial documents before 2014 and the potential duplication caused by different judicial procedures within the same case, this study further restricts the filing period and trial procedure. Specifically, the screening criteria were set as follows: filing date from January 1, 2014 to December 31, 2020; cause of action as medical malpractice liability disputes; trial procedure as first instance; document type as all; case type as all; and court level as all. Based on these criteria, a total of 39,809 medical malpractice liability dispute cases filed and adjudicated across provinces from 2014 to 2020 were collected.

This study also draws on the *China Health and Family Planning Statistical Yearbook* and the *China Health Statistics Yearbook*, which provide detailed province-level indicators on healthcare system performance and medical service provision. In addition, the *Statistical Yearbooks* of individual provinces are used to obtain province-level socioeconomic variables, including indicators of economic development, for the period 2012–2020.

Dependent variable. The dependent variable in this study is healthcare quality. The World Health Organization (WHO) defines healthcare quality as the degree to which healthcare services provided to individuals and populations increase the likelihood of desired health outcomes and are consistent with current evidence-based professional knowledge. This concept encompasses multiple dimensions, including effectiveness, safety, people-centeredness, timeliness, equity, and efficiency, and continues to evolve over time (24, 25). Although some studies have attempted to measure healthcare service performance or healthcare service levels at the provincial level in China, and have conducted extensive analyses using routine statistical indicators to construct composite indices or evaluate efficiency (26, 27), these indicators mainly reflect macro-level health system performance and cannot fully capture the multiple dimensions of healthcare quality described above. Therefore, this study follows the existing literature by using records of medical malpractice liability disputes as a proxy measure (28).

The use of medical malpractice liability dispute records as a proxy for healthcare quality is based on two considerations. First, these disputes usually involve medical injury, inappropriate diagnosis or treatment, delayed treatment, inadequate informed consent, and problems in medical record writing or management (29). They therefore capture relatively severe and externalized patient safety risks, process-related quality deficiencies, and patient-centered service problems that are difficult to observe through composite indices based mainly on routine statistical indicators. Second, in terms of data source, medical malpractice liability dispute records are generated through judicial procedures and constitute an external data source outside medical institutions (30). Compared with internal adverse event reporting systems or quality management data within healthcare institutions, litigation records do not fully depend on the active reporting of adverse events by medical institutions. They can therefore reduce, to some extent, the potential bias caused by underreporting within institutions and provide objective information for identifying healthcare quality problems from the perspectives of patient rights protection and judicial review.

Specifically, given that medical disputes are more likely to arise in the inpatient setting, this study constructs the incidence rate of medical malpractice liability disputes per 10,000 inpatient admissions by dividing the number of medical malpractice liability dispute cases in each province by the number of inpatient admissions in that province. Furthermore, because previous studies have commonly used indicators such as inpatient mortality, 30-day readmission, patient satisfaction, and average length of stay to assess healthcare quality (31), this study additionally includes the inpatient mortality rate as another measure of healthcare quality.

Independent variable. The key independent variable in this study is NCMR pilot. It is coded as 1 if a province had been designated as an NCMR pilot province in a given year, and 0 otherwise.

Because some time-invariant factors are absorbed by province fixed effects, this study follows the variable selection strategies and recommendations in the existing empirical literature on healthcare quality and healthcare reform, and mainly includes time-varying control variables in the domains of population, economy, healthcare, and education. Descriptive statistics for the relevant variables are reported in Table 1.

(1) *Population*. In terms of provincial demographic conditions, provinces with larger populations tend to face stronger demand for medical services, which may in turn affect the quality of healthcare provision. This study therefore uses the logarithm of the year-end resident population to measure provincial population size (32). In terms of population structure, provinces with a higher degree of population aging tend to have greater demand for medical services, which may in turn affect healthcare quality (33). This study therefore uses the proportion of the population aged 65 years and above in the total population to measure the degree of population aging in each province.
(2) *Economic development*. In terms of provincial economic conditions, provinces with higher levels of economic development generally have better-developed healthcare systems and are therefore more likely to provide higher-quality medical services (34). This study uses the logarithm of GDP per capita and the shares of the secondary and tertiary industries to measure the level of economic development and the economic structure of each province, respectively.
(3) *Medical resources*. In terms of the availability of medical resources across provinces, an insufficient supply of healthcare resources may place excessive operational pressure on medical institutions and increase the workload of medical staff, thereby adversely affecting healthcare quality (35). This study uses the logarithm of the number of beds in health institutions and the number of licensed physicians to measure the level of medical resources in each province.
(4) *Education*. From the perspective of human capital accumulation across provinces, provinces with greater public investment in education and higher levels of educational attainment are likely to have patients with better health literacy (36), medical staff with stronger risk awareness and management capacity, and a higher level of trust between patients and providers, all of which may affect healthcare quality (37). This study therefore uses the logarithm of local fiscal expenditure on education to measure the level of education in each province.

**Table 1:** Descriptive statistics of variables.

Variable type	Variable name	Definition	Mean	Standard deviation	Minimum	Maximum
Dependent variables	Medical malpractice dispute incidence rate	Incidence of medical malpractice liability dispute cases per 10,000 inpatient admissions	0.236	0.231	0	1.389
	Inpatient mortality rate	Inpatient mortality rate in healthcare institutions	0.418	0.328	0.05	1.7
Independent variable	NCMR pilot	0 = province had not yet started the pilot in that year; 1 = province had started the pilot	0.211	0.409	0	1
Control variables	Population size	Log of year-end resident population (10,000 persons)	8.132	0.842	5.753	9.443
	Regional GDP per capita	Log of regional GDP per capita (yuan)	10.82	0.424	9.85	12.009
	Share of secondary and tertiary industries	Share of output value of secondary and tertiary industries in regional GDP	0.500	0.087	0.163	0.655
	Number of beds in healthcare institutions	Log of number of beds in healthcare institutions (10,000 beds)	2.892	0.883	−0.174	4.201
	Number of licensed physicians	Log of number of licensed physicians (10,000 persons)	1.896	0.854	−1.238	3.301
	Local fiscal expenditure on education	Log of local fiscal expenditure on education (100 million yuan)	6.556	0.688	4.548	8.164
	Aging rate	Share of population aged 65 and above	0.106	0.025	0.050	0.174

### 3.2 Empirical strategies and model construction

This study primarily examines the impact of the NCMR pilot policy on healthcare quality. Because the NCMR pilot was implemented in different provinces in a staggered manner and has the characteristics of a quasi-natural experiment, this study employs a multi-period difference-in-differences (DID) model to identify the potential effects of the policy on healthcare quality before and after its implementation. The baseline regression model is specified as follows:


$$\;\;Y_{\mathrm{jt}}=\beta_{0}+\beta_{1}\text{Polic}y_{\mathrm{jt}}+\gamma X_{\mathrm{jt}}+\delta_{j}+\lambda_{t}+\varepsilon_{\mathrm{jt}}$$ (1)

Where j denotes the province and t denotes the year. $Y_{\mathrm{jt}}$ represents healthcare quality in province j in year t , measured primarily by two indicators: the medical malpractice dispute incidence rate and the inpatient mortality rate. The key explanatory variable, $\text{Polic}y_{\mathrm{jt}}$ , is a dummy variable indicating whether province j was designated as a NCMR pilot in year t . Its coefficient, $\beta_{1}$ , is the main parameter of interest and captures the effect of the NCMR policy on healthcare quality. $X_{\mathrm{jt}}\;$ denotes province-level control variables, $\delta_{j}\;$ denotes province fixed effects, $\lambda_{t}$ denotes year fixed effects, and $\varepsilon_{\mathrm{jt}}\;$ is the error term.

The use of a multi-period DID model requires that the NCMR pilot provinces and the non-pilot provinces follow similar trends prior to policy implementation, that is, the parallel trends assumption must hold. Because the timing of policy exposure differed across pilot provinces, this study constructs a set of dummy variables based on the relative timing of policy implementation in each pilot province and applies an event-study approach to test the parallel trends assumption in Equation (2), as follows:


$$\begin{array}{c} Y_{\mathrm{jt}}=\beta_{0}+\beta_{1}\mathrm{Pre}3_{\mathrm{jt}}+\beta_{2}\mathrm{Pre}2_{\mathrm{jt}}+\beta_{3}\mathrm{Pre}1_{\mathrm{jt}}+\beta_{4}\text{Curren}t_{\mathrm{jt}}\\ +\beta_{5}\text{Post}1_{\mathrm{jt}}+\beta_{6}\text{Post}2_{\mathrm{jt}}+\;\beta_{7}\text{Post}3_{\mathrm{jt}}+\beta_{8}\text{Post}4_{\mathrm{jt}}\\ +\beta_{9}\text{Post}5_{\mathrm{jt}}+\gamma X_{\mathrm{jt}}+\delta_{j}+\lambda_{t}+\varepsilon_{\mathrm{jt}} \end{array}$$ (2)

Where the time dummy variables capture the observations for each province in the n years before policy implementation, the year of implementation, and the n years after implementation, while the dummy variables for non-pilot provinces are set to 0. Because the sample period in this study spans 2012–2020, whereas the NCMR pilot was implemented in 2015 and 2016, only a subset of provinces has observations for the −4 period. To avoid multicollinearity, the dummy variable for the −4 period is therefore excluded. Finally, this study uses the time dummies for the −1 , −2 , and −3 periods prior to policy implementation as the reference periods for the parallel trends test, and focuses on the coefficients $\beta_{1}$ , $\beta_{2}$ , and $\beta_{3}$ . If all three coefficients are statistically insignificant, this indicates that there were no significant differences in healthcare quality between pilot and non-pilot provinces before the policy was implemented, and the parallel trends assumption cannot be rejected.

As a major national strategic initiative, the National Comprehensive Medical Reform (NCMR) pilot took into account interprovincial differences in economic development, medical insurance coverage, and other relevant conditions when selecting pilot provinces. We therefore argue that the pilot provinces are broadly representative at the national level. At the same time, the gradual rollout and deepening of the NCMR pilot coincided with a critical stage in China’s broader healthcare reform, during which other region-specific reform measures were introduced and may also have affected healthcare quality in pilot provinces, thereby confounding the estimated policy effect of the NCMR. Beyond this concern, potential endogeneity may also arise from baseline characteristics, omitted variables, measurement error, and heterogeneous treatment effects. To address these issues, this study conducts a series of robustness checks from the following eight aspects: (1) excluding the influence of other concurrent policies; (2) excluding the impact of the COVID-19 pandemic; (3) accounting for interference from baseline characteristics; (4) replacing the indicators of healthcare quality; (5) applying propensity score matching (PSM); (6) adjusting the treatment timing for the second-batch pilot provinces; (7) conducting a placebo test; and (8) examining staggered-adoption bias and treatment-effect heterogeneity through the Bacon decomposition and heterogeneity-robust DID estimators.

## 4 Empirical results and analyses

### 4.1 Benchmark regression

Table 2 reports the baseline regression results. The findings show that significant differences exist between the pilot and non-pilot provinces in both the incidence of medical malpractice disputes and inpatient mortality, with the estimated coefficients being significantly negative at the 5 and 1% levels, respectively. Specifically, relative to non-pilot provinces, the NCMR pilot is associated with a reduction of 0.088 medical malpractice dispute cases per 10,000 inpatient admissions, which corresponds to a decline of approximately 37.3% relative to the sample mean. The inpatient mortality rate is reduced by 0.5 per thousand, equivalent to a decline of about 11.9% relative to its mean value. Taken together, these results indicate that the NCMR pilot policy significantly reduced the incidence of medical disputes and inpatient mortality, thereby effectively improving healthcare quality.

**Table 2:** Impact of the NCMR pilot on healthcare quality.

Variable	(1)	(2)
	Medical malpractice dispute incidence rate	Inpatient mortality rate
NCMR pilot	−0.088^**^ (0.037)	−0.050^***^ (0.013)
Constant	−10.342 (8.318)	11.015^***^ (2.993)
Control variables	Yes	Yes
Year fixed effects	Yes	Yes
Province fixed effects	Yes	Yes
Observations	217	279
*R*^2^	0.812	0.973

Because complete data on medical malpractice liability disputes are not publicly available for the earlier years, column (1) uses the 2014–2020 sample. Values in parentheses are robust standard errors. *, **, and *** indicate significance at the 10, 5, and 1% levels, respectively. Unless otherwise stated, the same applies to all subsequent tables.

### 4.2 Robustness tests

#### 4.2.1 Parallel trends test and dynamic effects

**Figure 1:**
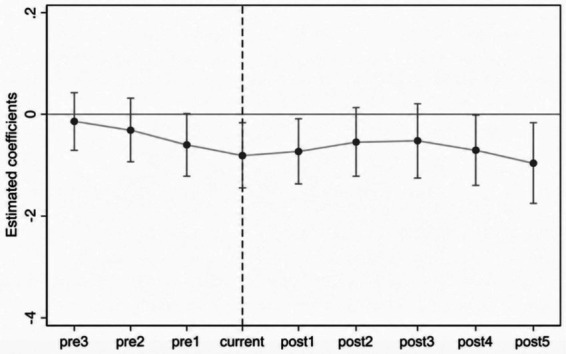
Parallel trends test and dynamic effects.

Due to data availability, the parallel trends test mainly uses the inpatient mortality rate. The medical malpractice liability dispute data from China Judgments Online are available only from 2014 onward, while the first-batch NCMR pilot provinces entered the reform in 2015. This leaves an insufficient pre-policy period for conducting a complete event-study test for the dispute variable. Therefore, Figure 1 reports the event-study estimates for inpatient mortality, for which a longer pre-policy period is available.

Figure 1 plots the estimated coefficients of $\beta_{k}$ for the inpatient mortality rate in Equation (2), together with their 95% confidence intervals. As shown in the figure, the estimated coefficients on the relative-time dummies prior to policy implementation are all statistically insignificant and relatively small in magnitude. In other words, the null hypothesis that $\beta_{k}=0\;$ cannot be rejected for the pre-policy periods, indicating that there were no significant differences in healthcare quality between the pilot and non-pilot provinces before the implementation of the NCMR policy. This finding therefore supports the parallel trends assumption.

In addition, the event-study results also provide supplementary evidence for understanding the dynamic effects of the NCMR policy. After policy implementation, the estimated coefficients are generally negative, which is consistent with the baseline regression results. Negative effects already appear in the implementation year and the early post-policy period. The effects weaken in the third and fourth post-policy years, while the negative effects become more pronounced again in the longer post-policy period.

#### 4.2.2 Excluding the influence of other concurrent policies

During the study period, several local or regional healthcare reforms were also promoted in China, including public hospital comprehensive reform and family doctor contract services. These policies may have affected quality-related outcomes, such as the incidence of medical malpractice liability disputes and inpatient mortality, by changing patients’ healthcare-seeking patterns, hospital operating mechanisms, and the allocation of medical resources. Therefore, they may confound the estimated effects of the NCMR pilot. Following the existing literature (38), this study further incorporates province-specific time trends into the baseline regression in order to capture the influence of regional healthcare reform policies on the outcomes. The results reported in columns (1) and (2) of Table 3 show that after allowing provinces to follow different time trends, the estimated effects of the NCMR pilot remain consistent with the baseline results.

**Table 3:** Robustness test A: Excluding regional policies, COVID-19, and baseline characteristics.

Variable	Excluding regional policy effects		Excluding COVID-19 effects		Controlling for baseline characteristics	
	(1)	(2)	(3)	(4)	(5)	(6)
	Medical malpractice dispute incidence rate	Inpatient mortality rate	Medical malpractice dispute incidence rate	Inpatient mortality rate	Medical malpractice dispute incidence rate	Inpatient mortality rate
NCMR pilot	−0.002^***^ (0.001)	−0.057^***^ (0.013)	−0.073^**^ (0.036)	−0.053^***^ (0.013)	−0.082^*^ (0.044)	−0.083^***^ (0.017)
Constant	−0.434^**^ (0.190)	10.568^***^ (2.917)	−16.172^**^ (7.979)	7.670^***^ (2.534)	0.805 (1.793)	−1.663^*^ (0.930)
Province-specific time trends	Yes	Yes	No	No	No	No
Control variables	Yes	Yes	Yes	Yes	No	No
Baseline controls × time trends	No	No	No	No	Yes	Yes
Year fixed effects	Yes	Yes	Yes	Yes	Yes	Yes
Province fixed effects	Yes	Yes	Yes	Yes	Yes	Yes
Observations	217	279	186	248	217	279
*R*^2^	0.859	0.976	0.838	0.976	0.865	0.981

#### 4.2.3 Excluding the influence of public health emergencies

Since 2020, the COVID-19 pandemic has severely disrupted the orderly operation and development of China’s healthcare system. To rule out the possibility that the pandemic may confound our core findings, this study excludes the 2020 observations and re-estimates the baseline regression. The results, reported in columns (3) and (4) of Table 3, are broadly consistent with the baseline estimates. This suggests that the COVID-19 shock does not materially affect our main conclusions, which therefore remain reliable.

#### 4.2.4 Controlling for baseline characteristics

Considering that differences in provinces’ baseline characteristics, as well as the potential effects of the policy on related factors, may interfere with causal identification (39), this study further tests the robustness of the results by incorporating time trends interacted with baseline control variables. As shown in columns (5) and (6) of Table 3, after replacing the control variables with interactions between the baseline values of the covariates and year dummies, the results remain consistent with the baseline regression. This provides further support for the robustness of our findings.

#### 4.2.5 Alternative measures of healthcare quality

Because provinces differ objectively in terms of economic development, natural environment, and other contextual factors, their disease profiles, population health status, and patterns of healthcare utilization may also vary accordingly (40, 41). Therefore, the medical malpractice dispute incidence rate measured using inpatient admissions may be affected by the omitted factors discussed above. To address this concern, this study replaces the number of inpatient admissions with two alternative denominators to remeasure the incidence of medical malpractice disputes: (1) total outpatient and emergency visits (10,000 persons) and (2) total healthcare visits (10,000 persons). The results are reported in Table 4. As shown in columns (1) and (2), the estimated coefficients remain negative and statistically significant at least at the 5% level across these alternative measures, indicating that the baseline results remain robust.

**Table 4:** Robustness test B: Alternative healthcare quality indicators, PSM and Delayed Policy Implementation.

Variable	Alternative healthcare quality indicators		PSM		Delayed Policy Implementation	
	(1)	(2)	(3)	(4)	(5)	(6)
	Medical malpractice dispute incidence rate (per 10,000 outpatient and emergency visits)	Medical malpractice dispute incidence rate (per 10,000 healthcare visits)	Medical malpractice dispute incidence rate	Inpatient mortality rate	Medical malpractice dispute incidence rate	Inpatient mortality rate
NCMR pilot	−0.097^**^	−0.002^**^	−0.079^**^	−0.049^***^		
NCMR pilot (adjusted timing)	(0.041)	(0.001)	(0.038)	(0.013)	−0.089^***^ (0.033)	−0.033^**^ (0.013)
Constant	−4.835 (4.208)	−0.166 (0.117)	−14.080^*^ (7.920)	10.302^***^ (2.851)	−10.909 (8.555)	11.840^***^ (3.058)
Control variables	Yes	Yes	Yes	Yes	Yes	Yes
Year fixed effects	Yes	Yes	Yes	Yes	Yes	Yes
Province fixed effects	Yes	Yes	Yes	Yes	Yes	Yes
Observations	217	217	206	268	217	279
*R*^2^	0.823	0.851	0.837	0.974	0.813	0.973

#### 4.2.6 Propensity score matching

To mitigate potential selection bias arising from observable characteristics, this study employs propensity score matching. Specifically, whether a province was designated as an NCMR pilot province is treated as a binary dependent variable, while the control variables are included as covariates in a logit model to estimate the propensity scores. Matching is then conducted using kernel matching with the default kernel function and bandwidth. After excluding observations that cannot be successfully matched, the policy effect is subsequently estimated using the matched sample.^1^ The results reported in columns (3) and (4) of Table 4 show that the coefficient on the key explanatory variable remains significantly negative, suggesting that sample selection bias has only a limited effect on the main findings of this study.

#### 4.2.7 Accounting for delayed policy implementation

In the baseline model, the second-batch NCMR pilot provinces are coded as treated from 2016 onward. However, the official designation year may not fully correspond to the first year of effective local implementation. The second batch was announced at the central level in May 2016, while local implementation plans were generally issued between May and July 2016. Therefore, in an annual panel setting, coding 2016 as the first treatment year may prematurely assign these provinces to the treated status. To address this concern, we recoded the second-batch pilot provinces as treated from 2017 onward and re-estimated the baseline model. The results in Table 4 remain significantly negative for both the medical malpractice dispute incidence rate and inpatient mortality, indicating that the main findings are robust.

#### 4.2.8 Placebo test

**Figure 2:**
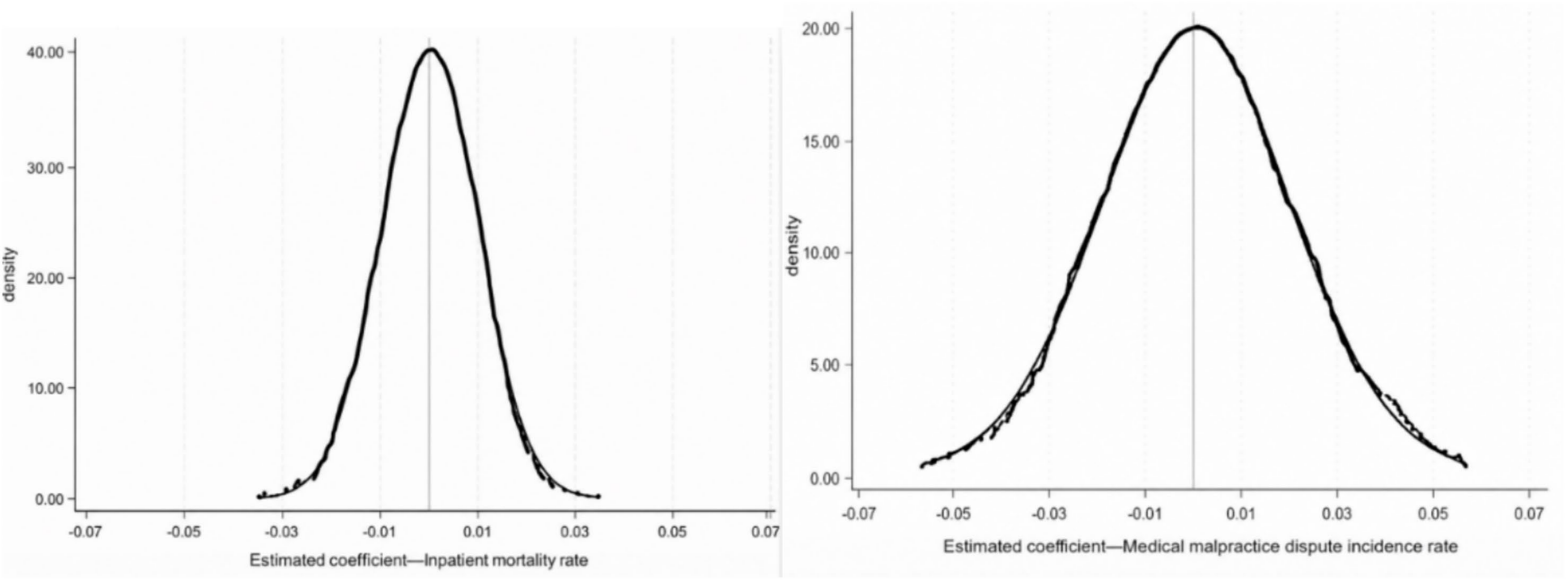
Placebo test.

Furthermore, this study conducts a placebo test to rule out the influence of unobserved factors. Specifically, provinces are randomly assigned to the treatment group, while the remaining provinces are assigned to the control group. Based on these randomly generated samples, the regression is repeated 1,000 times. Figure 2 presents the distribution of the estimated coefficients. As shown in the figure, the coefficients and *p*-values obtained from the randomly assigned pseudo-pilot groups are approximately normally distributed, and the estimated coefficients are all close to zero. This suggests that unobserved factors are unlikely to drive the estimated results.

#### 4.2.9 Heterogeneity-robust DID estimation

Considering that provinces entered the NCMR pilot at different points in time, the use of a multi-period DID model estimated with two-way fixed effects (TWFE) may be subject to potential bias arising from heterogeneous treatment effects and negative weighting (42). To diagnose this potential bias, we employ the Bacon decomposition proposed by Goodman-Bacon. Following the approach of Margaryan (43), we include only two-way fixed effects in the decomposition in order to avoid the influence of additional control variables, and then decompose the estimated coefficients and weights across groups with different policy implementation timings. The results of the Bacon decomposition are reported in Table 5. As indicated by the weight column, the estimated effect in the baseline regression is driven primarily by the true treatment effect (with a weight of 91.8%), suggesting that the results are only minimally affected by treatment-effect heterogeneity or time variation.

**Table 5:** Robustness test C: Bacon decomposition results.

Comparison type	Medical malpractice dispute incidence rate		Inpatient mortality rate	
	Coefficient	Weight	Coefficient	Weight
Earlier-treated vs. later-treated	0.006	0.014	0.014	0.014
Later-treated vs. earlier-treated	−0.018	0.068	−0.039	0.068
Time-varying treated vs. never-treated	−0.007	0.918	−0.055	0.918
Overall treatment effect	−0.008	/	−0.053	/

**Figure 3:**
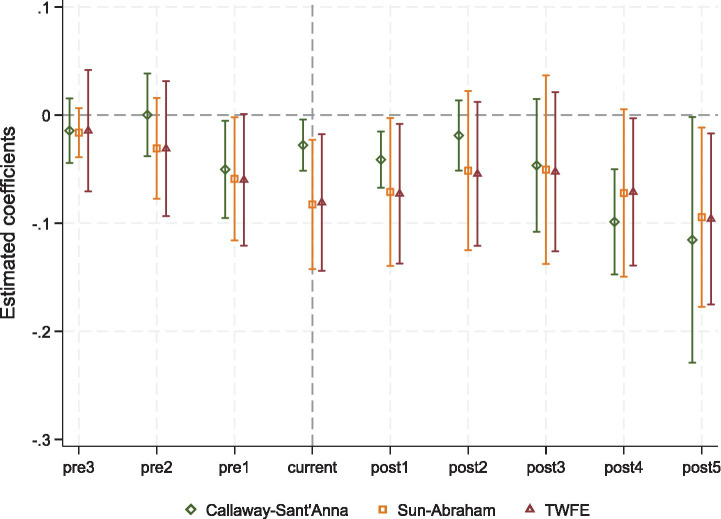
Heterogeneity-Robust DID Estimation.

Furthermore, to address potential bias arising from treatment-effect heterogeneity under staggered treatment adoption, this study additionally employed two heterogeneity-robust DID estimators: the group-time average treatment effect estimator proposed by Callaway and Sant’Anna and the interaction-weighted estimator proposed by Sun and Abraham (39, 44). Building on the event-study specification in Equation (2) and the baseline dynamic estimates shown in Figures 1, 3 compares the estimates obtained from these two heterogeneity-robust estimators with the TWFE event-study estimates. The results show similar pre-treatment patterns and broadly consistent post-treatment effect directions across the three estimators, suggesting that the main conclusions remain valid after accounting for potential bias caused by heterogeneous treatment effects.

### 4.3 Mechanism analysis

This study focuses on pharmaceutical pricing reform as a core policy component and empirically examines its potential mechanism through the optimization of the medical expenditure structure. Tables 6, 7 report the effects of the NCMR policy on per-visit outpatient and inpatient expenditures and their composition, respectively. The results in column (1) of both tables show that the policy significantly reduced total expenditure per inpatient admission, but did not significantly reduce total expenditure per outpatient visit. The results in column (2) indicate that the NCMR pilot significantly reduced drug expenditure per outpatient visit and per inpatient admission, suggesting that measures such as the zero-markup drug policy and centralized procurement may have produced the expected cost-containment effects. Furthermore, the results in columns (3)–(5) show changes in the expenditure structure that are consistent with the mechanism proposed in this study. Specifically, the policy significantly reduced the share of drug expenditure while increasing the share of service fees, which primarily reflect the labor value of medical staff. At the same time, we also observed a concurrent increase in the share of examination fees. Overall, these findings suggest that the NCMR may have reshaped medical expenditure structures and provider incentives by reducing healthcare institutions’ dependence on drug-related revenue and increasing the relative importance of service-based income. This mechanism provides a possible explanation for understanding improvements in healthcare quality.

**Table 6:** Mechanism analysis A: Impact of the NCMR pilot on per-visit outpatient expenditures and expenditure structure.

Variable	(1)	(2)	(3)	(4)	(5)
	ln(total expenditure per outpatient visit)	ln(drug expenditure per outpatient visit)	Share of outpatient drug expenditure	Share of outpatient examination fees	Share of outpatient service fees
NCMR pilot	0.002 (0.012)	−0.026^*^ (0.016)	−0.013^***^ (0.004)	0.004^*^ (0.002)	0.008^**^ (0.004)
Constant	6.194^***^ (1.828)	4.641^**^ (2.143)	0.549 (0.636)	0.966^*^ (0.498)	−0.515 (0.504)
Control variables	Yes	Yes	Yes	Yes	Yes
Year fixed effects	Yes	Yes	Yes	Yes	Yes
Province fixed effects	Yes	Yes	Yes	Yes	Yes
Observations	279	279	279	279	279
*R*^2^	0.980	0.971	0.941	0.953	0.914

**Table 7:** Mechanism analysis B: Impact of the NCMR pilot on per-admission inpatient expenditures and expenditure structure.

Variable	(1)	(2)	(3)	(4)	(5)
	ln(total expenditure per inpatient admission)	ln(drug expenditure per inpatient admission)	Share of inpatient drug expenditure	Share of inpatient examination fees	Share of inpatient service fees
NCMR pilot	−0.035^***^ (0.011)	−0.062^***^ (0.016)	−0.012^***^ (0.004)	0.003^**^ (0.001)	0.008^***^ (0.003)
Constant	8.960^***^ (1.836)	8.471^***^ (2.294)	−1.130^**^ (0.467)	0.408^**^ (0.185)	1.693^***^ (0.414)
Control variables	Yes	Yes	Yes	Yes	Yes
Year fixed effects	Yes	Yes	Yes	Yes	Yes
Province fixed effects	Yes	Yes	Yes	Yes	Yes
Observations	279	279	279	279	279
*R*^2^	0.985	0.967	0.966	0.931	0.956

## 5 Discussion

Healthcare quality is an important objective of deepening healthcare system reform and a key dimension for evaluating the performance of comprehensive medical reform. Using panel data from 31 provinces in China, this study employs a multi-period DID approach to assess the impact of the NCMR pilot on healthcare quality. The findings show that the NCMR pilot was associated with reductions in the incidence of medical malpractice liability disputes and inpatient mortality, and these results remain robust across a series of robustness checks. The mechanism analysis further shows that the NCMR pilot was accompanied by a decline in the share of drug expenditure and an increase in the share of service fees. This change in expenditure structure implies a reshaping of healthcare institutions’ revenue sources and provider incentive mechanisms, providing a possible explanatory pathway for understanding improvements in healthcare quality. However, this study also finds that the policy significantly increased the share of examination expenditures among both outpatient and inpatient services.

Compared with the existing literature on the effects of the NCMR, this study further extends the dimensions through which reform performance can be evaluated. Previous studies have mainly assessed the effects of the NCMR in terms of reducing medical burden, improving access to care, and promoting healthcare utilization (12). In contrast, this study uses two quality-related indicators, namely medical malpractice liability disputes and inpatient mortality, to provide additional empirical evidence on healthcare quality and patient safety. At the same time, this study innovatively uses CJO medical malpractice liability dispute records to measure healthcare quality at the provincial level. This extends the existing approach, which has mainly used judgment documents to conduct textual analyses of medical injury, doctor–patient relations, and their influencing factors, and provides a new pathway for evaluating health policy effects using external judicial data (45, 46). Furthermore, this study’s attempt to understand the potential quality-related effects of the NCMR from the perspective of medical expenditure restructuring also resonates with related macro-level research. Existing studies suggest that health expenditure may affect economic growth by improving human capital, labor productivity, and health welfare, but such effects depend not only on the scale of spending, but also on expenditure structure, resource allocation efficiency, and the organizational and financing arrangements of the health system (47, 48). From this perspective, the NCMR’s effects on the shares of drug expenditure, service fees, and examination expenditure not only reflect its cost-containment effects, but also reveal the broader significance of medical expenditure restructuring and health system incentive adjustment. More specifically, the value of comprehensive medical reform should not be understood merely as reducing medical costs, but should also be interpreted within a broader framework of optimizing health resource allocation, improving healthcare quality, and thereby strengthening the capacity for health capital formation. Notably, this study finds that the share of examination expenditures increased significantly among both outpatient and inpatient services, which is consistent with previous studies (16, 17). In the absence of sufficient supporting measures and regulatory coordination, healthcare institutions may still compensate for the decline in drug-related revenue by increasing examination fees, material charges, or the frequency of testing. Therefore, while reducing dependence on drug-related revenue, the NCMR also needs to guard against cost-containment pressures and potential quality risks associated with alternative revenue sources such as examination and testing.

Based on these findings, future reform should continue to reduce medical burden and expand access to care, while placing greater emphasis on sustained quality improvement through policy coordination. On the one hand, stronger coordination among medical care, medical insurance, and pharmaceuticals is needed, so that health insurance payment, pharmaceutical pricing governance, patient health outcomes, and healthcare quality can be more closely linked, thereby forming incentive and accountability mechanisms oriented toward health outcomes and quality improvement. On the other hand, continued efforts are needed to optimize the pricing structure of medical services and to remain alert to healthcare institutions’ reliance on examination and testing as alternative revenue sources after drug-related revenue is compressed. To this end, it is necessary to more clearly distinguish the technical labor value from material consumption costs in examination and testing items, improve a pricing system that better reflects the technical labor value of healthcare professionals, and simultaneously adjust government compensation mechanisms and hospitals’ internal remuneration systems. Aligning external payment rules with internal incentive mechanisms can provide a more sustainable institutional foundation for standardizing clinical practice and improving healthcare quality.

## 6 Limitations and future research

This study has several limitations. First, the quality indicator constructed from litigation materials should be interpreted with caution. Medical malpractice liability disputes are not determined solely by clinical quality; they may also be affected by regional rule-of-law conditions, patients’ legal literacy, socioeconomic status, and willingness to litigate. If these factors are associated with the selection of NCMR pilot provinces or the timing of entry into the pilot, the estimates may partly reflect changes in litigation behavior or judicial accessibility rather than healthcare quality itself. In addition, healthcare quality is multidimensional, while the indicators used in this study mainly capture patient safety and selected clinical outcomes, and cannot fully reflect dimensions such as timeliness and efficiency.

Second, the DID identification relies to some extent on the stable unit treatment value assumption. However, because the NCMR was implemented as a pilot reform, practices developed in pilot provinces may have diffused to non-pilot provinces through policy learning or regional coordination. Such spillovers may compress differences between pilot and non-pilot provinces and lead to conservative estimates. Conversely, if pilot provinces attracted physicians, patients, or other healthcare resources and generated resource-siphoning effects, the direction of bias may be more complex. Future research could use spatial DID or more granular data to examine policy spillover effects.

Third, this study is based on province-level panel data, and the estimates reflect average effects at the provincial level. Such measures may mask heterogeneity across areas, healthcare institutions, and patient groups within provinces. For example, declines in medical malpractice disputes or inpatient mortality may mainly reflect improvements in tertiary hospitals or high-level medical institutions, rather than equivalent improvements in primary care institutions or rural areas. Future studies could use prefecture-level, hospital-level, or patient-level data to examine the heterogeneous effects of the NCMR.

Fourth, this study is limited by the time span of the data. Because the medical malpractice dispute data were obtained from China Judgments Online, the sample ends in 2020. After 2021, the number of publicly available documents on this platform dropped sharply, possibly due to changes in disclosure rules, platform inclusion mechanisms, or other factors unrelated to healthcare quality. Including data from 2021 onward may therefore introduce additional measurement error. Moreover, CJO data are available only from 2014 onward, while the first batch of NCMR pilot provinces entered the policy period in 2015, leaving a short pre-policy period for the litigation-based quality indicator. Although the test based on inpatient mortality provides preliminary support for the parallel trends assumption, the pre-policy trend evidence for the litigation-based indicator remains limited. Future research should further update and validate these findings when more stable and complete data become available.

## Data Availability

The raw data supporting the conclusions of this article will be made available by the authors, without undue reservation.

## Footnotes

1 Considering potential differences across matching algorithms, we further implemented nearest-neighbor matching and caliper matching to enhance the reliability of the robustness checks. The results obtained from these two matching methods were consistent with those based on kernel matching. Owing to space constraints, these results are not reported here but are available from the authors upon request.
